# Antipsychotic prescribing patterns in Australia: a retrospective analysis

**DOI:** 10.1186/s12888-022-03755-z

**Published:** 2022-02-12

**Authors:** Nagesh Pai, Mustafa Acar, Prabhjot Juneja, Mahsa Hosseini Kouhkamari, Sinthuja Siva, Judy Mullan

**Affiliations:** 1 School of Medicine, University of Wollongong (UOW), Wollongong, Australia; 2 Illawarra Shoalhaven Local Health District (ISLHD), Wollongong, Australia; 3 Janssen-Cilag Pty Ltd, North Ryde, NSW Australia; 4 Prospection, Redfern, NSW Australia

**Keywords:** Schizophrenia, Persistence, Long-acting antipsychotic(s), Therapeutic relationship, Oral antipsychotic(s), Prescribing patterns, Clinical guidelines

## Abstract

**Background:**

To examine real-world patterns of antipsychotic use in patients with schizophrenia Australia.

**Methods:**

This retrospective cohort analysis was conducted using the Australian Commonwealth Department of Human Services Pharmaceutical Benefits Scheme (PBS) 10% sample data. Included data were for patients aged 16-years or older who initiated treatment for the first time with a PBS-reimbursed antipsychotic medication for schizophrenia between July 2013 and September 2017. Patterns of treatment usage were summarised descriptively. Differences in prescribing patterns by age and prescribing year were reported. Treatment persistence was estimated using Kaplan-Meier methods, with differences explored using log-rank tests. Values of p < 0.05 were considered statistically significant.

**Results:**

6,740 patients, representing 8,249 non-unique patients, received prescriptions for antipsychotic medications. Patients were aged 16 years to over 85 years (54.5% were < 55 years) and two-thirds of patients were male (61%). The majority of treatment episodes (62%, n = 5,139/8,249) were prescribed an atypical oral antipsychotic. Typical long-acting antipsychotic therapies (LATs) were prescribed 19% of the treatment episodes (n = 1,608/8,249. There was a small increase in prescribing of atypical LAT and typical LAT and a small decrease in atypical oral and clozapine prescribing over the study period. Treatment persistence was greatest in patients treated with clozapine, than in those treated with atypical LATs.

**Conclusions:**

While the majority of patients receive atypical antipsychotic medications, one in five continue to use older typical LAT therapies. Patient age and time on therapy may be associated with choice of therapy. Persistence to atypical LAT therapy is better than for other treatment modalities in this real-world cohort.

**Supplementary Information:**

The online version contains supplementary material available at 10.1186/s12888-022-03755-z.

## Background

Schizophrenia is one of the top ten contributors to the global burden of disease and disability, affecting 20 million people globally [1]. Approximately 1% of people worldwide experience schizophrenia during their lifetime [2]. An Australian national survey in 2010 reported over 30,000 adults aged between 18 and 64 years of age had a diagnosis of schizophrenia [3]. The highest prevalence is in the 25 to 54 year old age group, thus leading to significant economic losses [4].

Antipsychotic medication is the mainstay of treatment for schizophrenia. Pharmacological therapy is often employed along with psychosocial interventions, such as cognitive behavioural therapy, family intervention and social skills training, with recent research suggesting that both forms of therapy can help improve patient prognosis if offered as early interventions [5]. However, each individual’s response to antipsychotic medication treatment differs and response cannot be predicted, which necessitates a trial-and-error treatment choice strategy [6]. There are a number of different antipsychotic medications available including typical oral and injectable medications, and atypical oral and injectable medications. Despite these treatments, some patients remain treatment-resistant. Clozapine remains the drug of choice in treatment-resistant schizophrenia [7]. Other treatments can be useful, such as electroconvulsive therapy (ECT).

 The Royal Australian and New Zealand College of Psychiatrists (RANZCP) guidelines on the treatment of schizophrenia, advocate a ‘start low, go slow’ policy for people with treatment naïve schizophrenia. Atypical, or second generation, antipsychotic agents are recommended as the first-line treatment choice in treatment naïve patients [2]. Patients in the acute phase of schizophrenia may be prescribed atypical oral antipsychotics and switched to atypical long-acting therapy (LAT), for example paliperidone palmitate, aripiprazole long-acting injection, olanzapine depot injection or risperidone long-acting injection, once stable [2].

The benefits of continued antipsychotic treatment for relapse prevention are well-known, such as reduction of risk of structural brain damage and treatment resistance and maintenance of social functioning [8, 9], although the role of antipsychotics in structural brain damage is controversial [10, 11]. The risk of relapse when receiving continuous antipsychotic medication is approximately one-third of that on placebo [12]. Therefore, good persistence to treatment is paramount.

There are potential clinical benefits and cost-savings for the early use of LAT therapies compared to oral antipsychotic agents [13, 14]. These include lower rates of relapse and hospitalisations, reduced schizophrenia-related comorbidities and decreased use of healthcare resources [13]. A meta-analysis of mirror image studies, which compare a period of oral antipsychotic treatment with a subsequent period of LAT treatment for the same patients, reflecting the population of patients treated with LAT therapy in real clinical practice, have reported superiority of atypical LATs over oral antipsychotic medications (atypical or typical) in relapse prevention [14].

Little is known about real-world use of antipsychotics for the treatment of schizophrenia in Australia. Internationally, antipsychotic use has been studied in the general population, vulnerable patients (including veterans), the elderly, and in children and adolescents [15–21]. Real-world antipsychotic prescribing data have been compared to various medication algorithms and guidelines [22]; however, there is no such recent research involving antipsychotic prescribing in Australia.

## Aim

 The aim of this study was to investigate real-world antipsychotic medication use for schizophrenia in Australia and consider whether this was consistent with the Royal Australian and New Zealand College of Psychiatrist prescribing guidelines.

## Methods

This study was a retrospective, observational, non-interventional analysis of the Australian Department of Human Services Pharmaceutical Benefits Scheme (PBS) 10% sample. The PBS 10% sample is a de-identified systematic random sample of medication dispensed under an authority PBS prescription for 10% of the Australian population who were dispensed government (PBS) reimbursed medications. The dataset is made available to researchers and data custodians to answer specific research questions [23]. Informed consent was waived by the Australian Government Department of Human Services External Request Evaluation Committee. This study and publication of subsequent results were approved by the Australian Government Department of Human Services External Request Evaluation Committee (RMS1280).

### Patients

Patients, aged 16 years or over on the date of their first prescription of an antipsychotic medication in the treatment of schizophrenia, in the sampling window (1 July 2013 to 30 September 2017) were included in this analysis. Patients were clinically diagnosed by their treating physician according to usual practice. This diagnosis was inferred using PBS item codes (see Supplemental text). Patients were included in the analysis if they initiated an antipsychotic medication between July 2013 and Supplementary Text. Patients were excluded if they had a prescription of an antipsychotic medication in the period 2006 to 30 June 2013 (to ensure that included patients were assumed to be treatment naïve, or first-line patients, and to prevent a potential bias where concessional patients were not included in the cohort prior to 2012; most patients with schizophrenia have concessional status); had a concomitant or prior prescription of an antidepressant therapy (as these patients were assumed to be patients with depression requiring augmentation therapy), or a mood stabiliser (amitriptyline, citalopram, desvenlafaxine, dosulepin, doxepin, duloxetine, escitalopram, fluoxetine, imipramine, mianserin, mirtazapine, moclobemide, nortriptyline, paroxetine, phenelzine, reboxetine, sertraline, tranylcypromine or venlafaxine, see PBS item codes in Supplemental text); or if they were 16 years of age or under at the time of initiation of the antipsychotic.

### Data extraction

Data on all reimbursed prescriptions (both general and concessional), sex, year of birth, state, date of claim, and item code were extracted. The prescribed drug name, quantity dispensed, drug class (oral typical, oral atypical, typical long-acting therapy, atypical long-acting therapy, clozapine) and drug type were inferred from the PBS item code and its corresponding authority code (see Table 1).

**Table 1:** Antipsychotics available in Australia defined by class system per the current study

Drug Class	Antipsychotic medication
Oral typical	chlorpromazine; periciazine; haloperidol
Oral atypical	aripiprazole; olanzapine; quetiapine; risperidone; ziprasidone; paliperidone; brexpiprazole; amisulpride; asenapine; lurasidone; ziprasidone
Typical long-acting therapy	flupenthixol; zuclopenthixol; haloperidol
Atypical long-acting therapy	paliperidone; aripiprazole; risperidone; olanzapine
Clozapine	clozapine oral tablets

The first antipsychotic prescription within the sampling window was considered the first-line of therapy (Treatment 1 within the study period). A patient progressed to the next line of therapy if they switched antipsychotic group, added on another antipsychotic and or antidepressant, lithium or antiepileptic. Treatment switching was defined as changing from one class of antipsychotic to another. Subsequent lines of therapy were similarly calculated. Patients who dispensed for a period of 90 or more days, more than one antipsychotic and / or had concurrently dispensed antidepressants, clozapine, lithium or an anti-epileptic/convulsant were assumed to have employed polypharmacy as part of their treatment strategy. Treatment persistence was defined as the time, in consecutive days, from the date of the initial prescription (at whichever line), to the date they switched to a different class of therapy (e.g. oral atypical to oral typical agent), or to the date after which there was a period of 6-months without a PBS prescription.

### Statistical analysis

No formal sample size calculation was performed, and all available data meeting the inclusion criteria and none of the exclusion criteria were included in the analysis. Patient characteristics and patterns of prescriptions were summarised descriptively. For all analyses by class, patients with multiple lines of therapy were included more than once, and as such, the percentage share of prescribing by drug class was calculated using the number of treatment episodes (n = 8,249), rather than the number of unique patients (n = 6,740). Treatment persistence was calculated using Kaplan-Meier methods, with differences in persistence by treatment type explored using pairwise comparisons of persistence at 12-months with atypical oral antipsychotics considered the reference value. Values of p < 0.05 were considered statistically significant. All analyses were conducted using Prospection’s proprietary PharmDash software (Prospection, Sydney, Australia).

## Results

A total of 6,740 unique patients (and 8,249 treatment episodes) in the PBS 10% sample received prescriptions for an antipsychotic therapy between July 2013 and September 2017. Patients were aged from 16 years to over 85 years with almost two-thirds of patients being males (62% vs. 38%, male vs. female). A slightly greater proportion of women than men received typical LATs (22% vs. 17%).

### Prescribing patterns

Prescribing patterns are presented in Table 2. Overall, 62% of treatment episodes (n = 5,139/8,249) received an atypical oral antipsychotic medication. Oral atypical antipsychotics were the most frequently prescribed antipsychotics in first-line patients (n = 4,883/6,872; 71%), with only a small proportion receiving atypical LATs (n = 173/6,872, 3%). The prescription of atypical LATs increased to 21% (n = 428/1,297) in subsequent lines of therapy (Table 2).

**Table 2:** Antipsychotic use by line of therapy

Prescribed antipsychotic	Any line of therapy, n(%)	First line, n(%)	Second line or subsequent lines, n(%)
Treatment episodes, n (%)^a^	8,249 (100%)	6,872 (100%)	2,013 (100%)
Unique patients, n	6,740	6,740	1,297
Atypical Oral	5,139 (62%)	4,883 (71%)	428 (21%)
Typical LAT	1,608 (19%)	1,146 (17%)	684 (34%)
Typical Oral	751 (9%)	511 (7%)	319 (16%)
Atypical LAT	541 (7%)	173 (3%)	428 (21%)
Clozapine	210 (3%)	159 (2%)	684 (34%)

^a^Note: Total number of non-unique patients is higher than unique patients due to polypharmacy and patients re-entering the data set on different treatments. The denominator for percentage calculations is the total number of non-unique patients

**Fig. 1:**
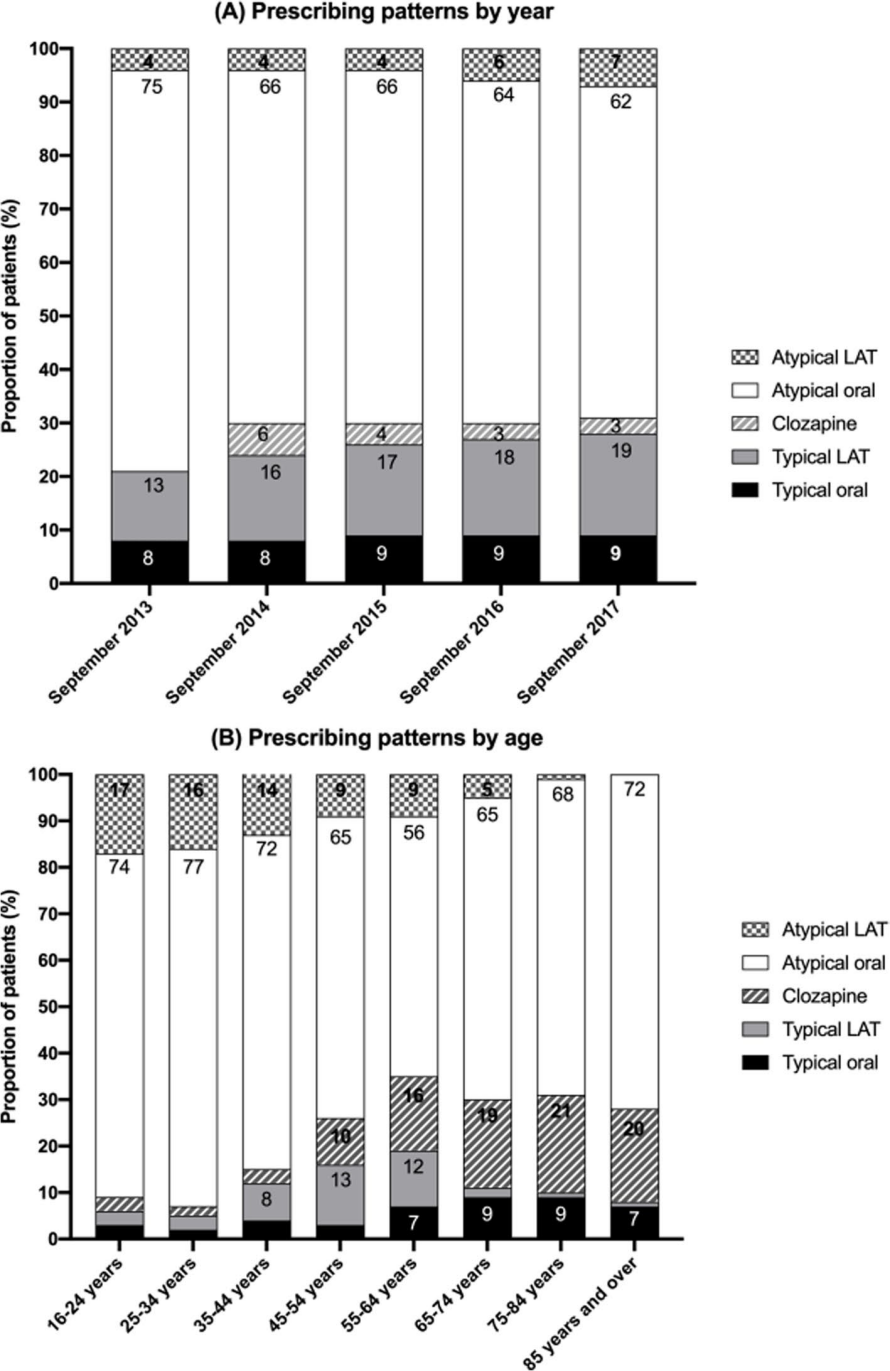
Prescribing patterns (**A**) by year (**B**) by age. Note: In Panel A, clozapine is not included in the 2013 figures as it was not available on the PBS. Percentages < 5 not annotated on the figure

Over the study period, there were increases in the proportion of atypical LAT prescriptions (4–7%), and in the proportion of typical LAT prescriptions (13–19%; Fig. 1 A). Higher rates of typical antipsychotic (LAT and oral) use and clozapine use were observed in patients aged 45 years and over (Fig. 1B). Atypical LATs were more frequently prescribed to younger patients (Fig. 1B)

### Polypharmacy

Overall, at the end of the study period, 25.1% of patients were on dual antipsychotic therapy and 2.8% were on triple antipsychotic therapy. Atypical oral therapy in the first-line was most commonly co-prescribed with an antidepressant (during the post-baseline period: 22.2% of combinations) and then with an antiepileptic medication (7.8% of combinations). Antidepressant medication was combined with first-line typical oral therapy in 17.3% of combinations, and with first-line atypical LATs, and first-line clozapine therapy in 7.9% and 7.6% of combinations, respectively. Clozapine in the first-line was most commonly prescribed with typical LAT therapy (27.1% of combinations). It should be noted that, in Australia, patients prescribed clozapine must be non-responsive or intolerant of other neuroleptic agents, and this is typically initiated within the hospital. Thus ‘first-line’ clozapine actually reflects patients for whom clozapine is the first treatment listed in the PBS dataset during the sampling window but does not reflect ‘first-line’ use of this agent. Patient numbers in the overall polypharmacy group are low.

### Antipsychotic treatment persistence

**Fig. 2:**
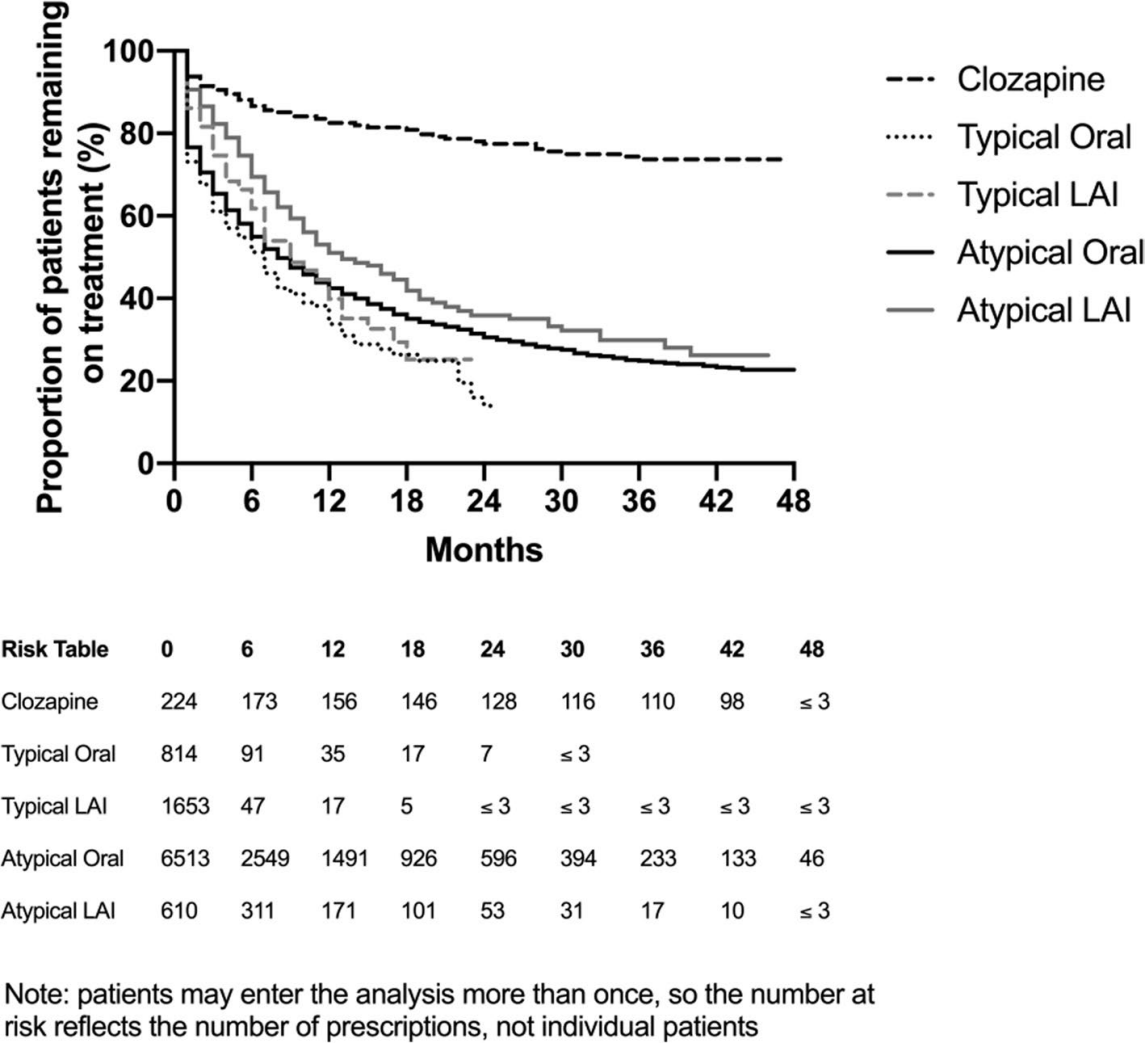
Treatment persistence by drug type

Median persistence to antipsychotic treatment, defined as the time from the initial prescription to the cessation of that treatment, was 7.9 months for atypical oral antipsychotics, 12.7 months for atypical LATs, 8.8 months for typical LATs, 6.2 months for typical oral antipsychotics. There was insufficient follow-up to calculate the median persistence to clozapine. In the overall cohort (all prescriptions of antipsychotics), when compared to atypical orals, atypical LATs and clozapine showed longer persistence (compared to atypical oral antipsychotics: atypical LAT RR 0.73, 95% CI 0.66 to 0.81, p < 0.01; typical oral antipsychotics RR 1.18, 95% CI 1.04 to 1.33, p < 0.01; typical LATs RR 0.68, 95% CI 0.61 to 0.75; clozapine 0.23, 95% CI 0.20 to 0.26, p < 0.01; Fig. 2).

### Treatment discontinuation

During the study period 3,447 patients discontinued therapy. Median time to retreatment for the discontinued cohort was 44 months. The median time to retreatment for patients restarting clozapine was 9 months. At the time of this analysis, 47% of patients had not returned to re-fill their antipsychotic prescription. For patients who recommenced treatment with antipsychotic medication after a six month treatment free period, 50% did so within ten months.

### Treatment switching

**Fig. 3:**
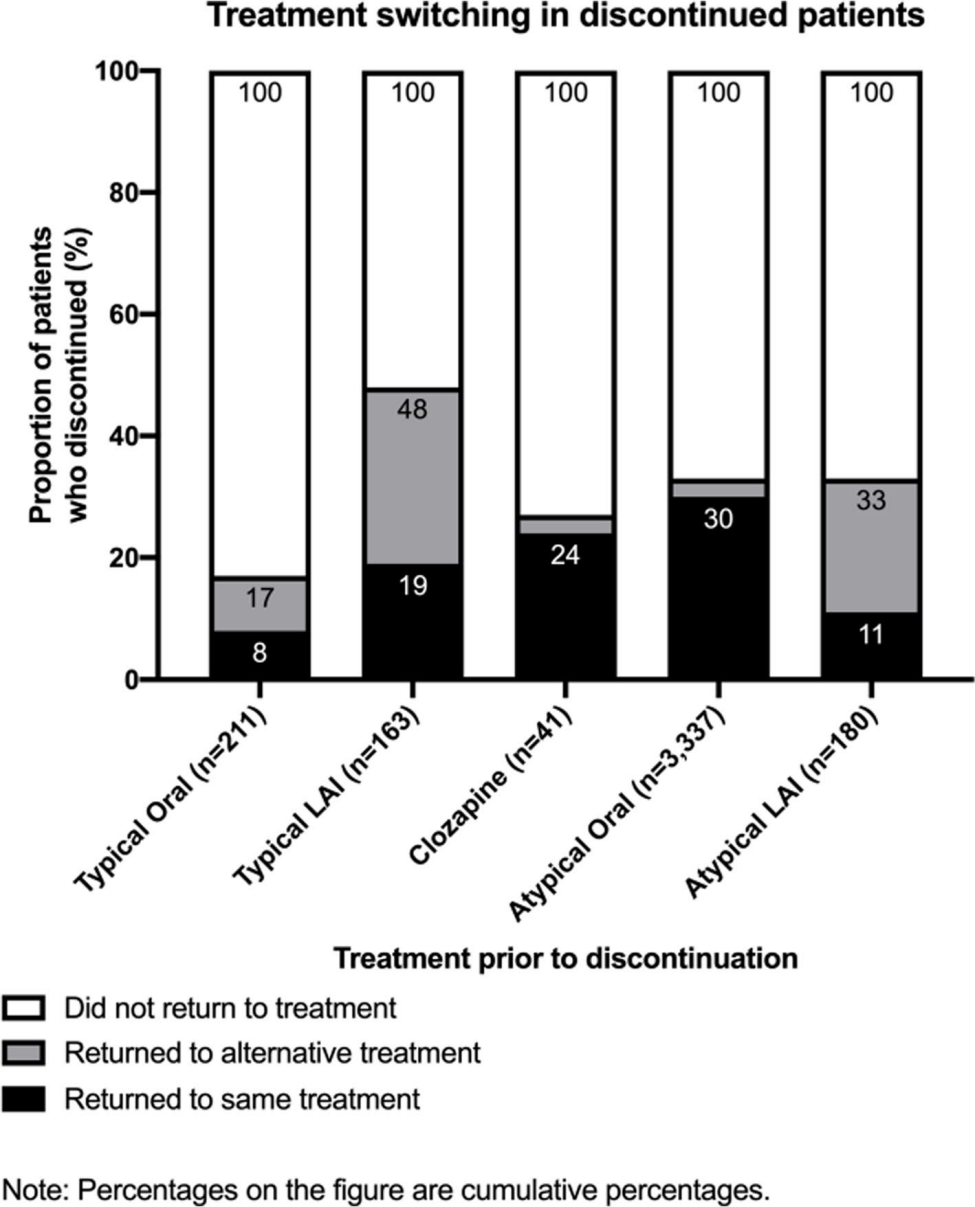
Percentage of patients returning to the same treatment, other treatment or did not return to treatment. Note: Percentages on the figure are cumulative percentages

For patients who previously discontinued atypical oral therapy, 30% of these patients recommenced the same treatment and 67% ceased treatment altogether. For patients who previously discontinued clozapine therapy, 24% of these patients recommenced the same treatment (Fig. 3). No patients initially receiving clozapine switched to a different treatment group. For all other drug classes, it was more common to recommence treatment with a drug from a different class to that initially prescribed. Overall, there were low rates of switching. Patients receiving typical orals switched more often; 31% had switched to typical LATs and 16% to atypical oral antipsychotic 12 months after initiating the typical oral antipsychotic (Table 3).

**Table 3:** Proportion of patients switching class at 6 and at 12 months

	Antipsychotic treatment group switched to									
Switch From	% of patient switches at 6 months of continuous antipsychotic therapy					% of patient switches at 12 months of continuous antipsychotic therapy				
	Atypical Oral	Atypical LAT	Typical Oral	Typical LAT	Clozapine	Atypical Oral	Atypical LAT	Typical Oral	Typical LAT	Clozapine
Atypical Oral	-	2%	1%	1%	<1%	-	3%	2%	2%	<1%
Atypical LAT	5%	-	0%	0%	0%	9%	-	0%	0%	0%
Typical Oral	11%	0%	-	26%	0%	16%	0%	-	31%	0%
Typical LAT	3%	1%	5%	-	0%	4%	1%	0%	-	7%
Clozapine	1%	0%	0%	1%	-	3%	0%	0%	1%	-

## Discussion

In line with RANZCP guidelines [2], atypical oral antipsychotics were the most commonly prescribed first-line antipsychotic medication. There was a trend over time to increased LAT use (both typical and atypical). Typical oral use has remained relatively steady, while the proportion of patients with atypical oral use has declined slightly.

Although there was increased LAT prescribing over the analysis period, the rate of typical LAT utilisation has increased at a faster rate than atypical LAT utilisation. This lower than expected uptake of atypical LAT therapy could be due to multiple factors: patient resistance to LAT in general due to a perception of coercion or stigma attached to the medication or a loss of control over treatment, clinician resistance due to limited knowledge and experience of LATs (especially newer agents such as paliperidone palmitate or aripiprazole long-acting injection) or a belief that patients will not accept their use [24–26]. It may reflect older patients returning to treatment on a molecule they are familiar with. However, information regarding these factors were outside the scope of the current study and would require further investigation. Among those who did receive treatment with an atypical LAT, there was a larger proportion of younger patients (adults aged up to 44 years) in the current study. Reasons for this are unknown, however it is possible that older patients who have previously been stabilised on a typical LATs are hesitant to switch therapies despite an improved side effect profile, such as reduced risk of tardive dyskinesia [27]. Alternatively, it may reflect suboptimal prescribing in the primary care setting [28].

Our data might suggest that Australian prescribers may need to consider a shift in prescribing, in order to align with the body of published data that shows improvements in clinical and economic outcomes with the use of atypical LATs [14]. This may require enhanced discussions with the patient about the available options, and the pros and cons of the various available LATs [24].

Data for the period 2006 to 2014 shows increased prevalence of antipsychotic medication use in Australia, and in particular increased use of atypical antipsychotics compared to typical antipsychotics [29]. By 2014 the atypical:typical antipsychotic prevalence ratio in Australia was 6:1 [29]. The increased use of atypical antipsychotics may be linked to expanded regulatory approval for indications, other than for the schizophrenia spectrum and the increase in prescribing for off-label indications [30]. Our examination, however, was restricted to those prescribed these agents for a diagnosis of schizophrenia. Examining our study data from 2014 onwards (excluding 2013 as clozapine was not included in this data at that time), there was a small decrease in the proportion of patients prescribed atypical antipsychotic oral medications. There are no clozapine data prior to 2015 in the PBS sample because the treatment was prescribed, and funded by hospitals whose pharmaceutical expenses are funded outside the PBS, under the authority of a hospital-based psychiatrist, with their prescription dispensed from the hospital pharmacy [31]. There has been an increase in LAT utilisation, however the utilisation of typical LATs has grown more than the utilisation of atypical LATs. As a consequence, the proportion of patients on atypical orals has subsequently reduced. This probably reflects increasing evidence for clinical benefits of LATs over their oral counterparts [14]. Some of the adverse effects of LATs may limit their use.

Rates of switching were low in our study. This is in line with Australian guidelines which recommend switching only in the context of intolerable side effects or lack of efficacy [2]. Switching patients can be risky, resulting in withdrawal syndromes, relapse or rebound [32].

The use of antipsychotic polypharmacy was prevalent at the end of the period studied, despite concerns about additional side effect burden being raised and there being little evidence for such practice [2], except for preventing emergency visits and rehospitalisations [33]. The most common polypharmacy was in combination with an antidepressant, so the indication for therapy may have instead been for comorbid depression. Similarly, high rates of polypharmacy among those with schizophrenia have been reported internationally [34–36]. These high rates of polypharmacy may be out of keeping with prescribing guidance internationally, where monotherapy is the standard of care for patients with schizophrenia [2, 37–39].

 Clozapine had the longest persistence to therapy, however, this may reflect the prescribing indication (treatment resistance) and guidelines which recommend its use for at least 12 months. Patients prescribed atypical LATs had longer persistence to therapy, followed by typical LATs. However, patients also may be more adherent to LAT use in general due to reduced frequency of adherence demands (fewer time points in which a decision to adhere to therapy is requested) [40]. Others have also reported improved adherence to therapy in patients prescribed LATs compared to those taking different oral antipsychotic in a real-world setting in the USA [41]. In that study, the median time to discontinuation of LAT was 6.4 months (95% CI 5.9 to 7.0 months), while persistence to oral antipsychotics was 4.0 months (95% 3.8 to 4.4 months), much shorter than that reported in our analysis. Data showing the longest persistence to clozapine compared with other treatment classes (83% of patients on therapy at 12 months, median not reached) is consistent with typical disease severity for which clozapine is indicated, guidelines suggesting a 12-month treatment trial and the level of external oversight in ensuring patients persist with treatment.

Limitations of this study include the following: the PBS 10% sample dataset does not include medicines supplied in hospitals, or those medications subsidised under the Repatriation PBS [42]. We excluded patients with concomitant antidepressant use prior to the analysis period in order to omit a pattern of prescribing that resembled augmentation in the management of major depressive disorder, as is recommended for some people with depression [43]. We did not consider prescribing outside of schizophrenia spectrum disorders, but for management of comorbid conditions such as insomnia, dementia, bipolar disorder or mood symptoms. We acknowledge use of PBS item codes to restrict our sample to patients with schizophrenia as a significant limitation given the clinical complexity that surrounds the treatment of schizophrenia, and that in some cases the PBS item code does not indicate a diagnosis as prescribing is unrestricted. Further our assumption that if a patient had not been treated for a period of 12 or more months as being ‘treatment naïve’ or ‘first line’ may have inflated the proportion of patients who were categorised as receiving typical LATs in the first line setting. It is not known if there were systematic differences in type of patients which may have affected the results of this study. We could not assess what clinical factors (e.g. comorbidities, disease severity) may contribute to the outcomes described, as these were not available in our dataset, therefore we do not know whether outcomes may vary by such factors. Reasons for discontinuation are not known, therefore, it is unclear if changes occurred due to side effects, lack of efficacy or other reasons. Finally, the PBS data only contains information on whether a treatment was dispensed or not, not the reason for that prescription. Thus, while LAT use has increased over time, and one could speculate this is due to a lack of adherence to oral medication, this cannot be assessed using the PBS data. This would be of interest for future research.

## Conclusions

While the majority of patients receive atypical antipsychotic medications, one in five continue to user older typical LAT therapies and 7% of the total cohort have access atypical LAT therapies. Patient age and time on therapy may be associated with choice of therapy. Persistence to atypical LAT therapy is better than for other treatment modalities in this real-world cohort. Further research should address why uptake of LATs is not as high as might be expected given their efficacy and tolerability with consideration given to improving education of prescribers and patients on the benefits of LAT. Other areas of future research include the rates of polypharmacy and discontinuation, which are both key issues for clinicians.

## Supplementary Information

**Additional file 1.**

**Additional file 2.**

## Data Availability

The data that support the findings of this study are available from the Australian Commonwealth Department of Human Services PBS 10% sample data, accessed via licence from Services Australia. For further information and to access the PBS 10% sample data see https://www.servicesaustralia.gov.au/organisations/about-us/reports-and-statistics/statistical-information-and-data#a2.

## Abbreviations

ECT: Electroconvulsive therapy
LAT: Long-acting antipsychotic therapies
PBS: Pharmaceutical Benefits Scheme
RANZCP: Royal Australian and New Zealand College of Psychiatrists
