# Supplementary material for: Antipsychotic prescribing patterns in Australia: a retrospective analysis
Source: BMC Psychiatry. 2022 Feb 12;22:110. doi: 10.1186/s12888-022-03755-z (PMC8840322; doi:10.1186/s12888-022-03755-z)
Supplement: Supplementary file 1 — Additional file 1. [file 12888_2022_3755_MOESM1_ESM.docx]

**SUPPLEMENTARY TEXT**

**PBS item codes for antipsychotics**

1001Q, 10219W, 10224D, 1024X, 10288L, 10289M, 10302F, 10341G, 10358E, 1037N, 1041T, 1042W, 1046C, 10526B, 10529E, 11066K, 11072R, 11085K, 11094X, 11184P, 11188W, 11189X, 11190Y, 11415T, 11422E, 11433R, 11869Q, 11872W, 11873X, 11874Y, 11877D, 11879F, 11881H, 11882J, 1195X, 1196Y, 1197B, 1199D, 1201F, 1842Y, 1846E, 2185B, 2186C, 2255Q, 2256R, 2257T, 2386N, 2761H, 2763K, 2765M, 2766N, 2767P, 2768Q, 2770T, 3052P, 3053Q, 3098C, 3169T, 3170W, 3171X, 3172Y, 3381Y, 3382B, 3384D, 3385E, 3455W, 3456X, 5100K, 5102M, 5103N, 5107T, 5109X, 5140M, 5141N, 5458G, 5626D, 5627E, 5628F, 5629G, 5630H, 6101D, 6102E, 6417R, 6418T, 8097E, 8100H, 8170B, 8185T, 8186W, 8187X, 8433W, 8434X, 8456C, 8457D, 8458E, 8580N, 8594H, 8595J, 8596K, 8717T, 8718W, 8719X, 8720Y, 8736T, 8780D, 8781E, 8782F, 8787L, 8788M, 8789N, 8790P, 8791Q, 8792R, 8794W, 8869T, 8870W, 8952E, 8953F, 9070J, 9071K, 9072L, 9073M, 9075P, 9076Q, 9079W, 9080X, 9140C, 9141D, 9142E, 9194X, 9202H, 9203J, 9204K, 9205L, 9293D, 9294E, 9295F, 9303P, or 9632Y

**PBS item codes for antidepressants**

2417F, 2418G, 2429W, 8702B, 8220P, 8703C, 1561E, 10245F, 9367B, 10231L, 10234P, 9366Y, 10241B, 1357K, 1358L, 1011F, 1013H, 1012G, 9155W, 9156X, 8700X, 9432K, 8849R, 8701Y, 9433L, 10181W, 9700M, 1434L, 8270G, 8174F, 8512B, 12581E, 2420J, 12113M, 2421K, 1627P, 1628Q, 8855C, 8856D, 8857E, 9365X, 8513C, 8883M, 1900B, 8003F, 2522R, 2523T, 2242B, 9197C, 2856H, 11713L, 12239E, 8583R, 12296E, 12317G, 2237R, 8837D, 12299H, 12305P, 2236Q, 8836C, 2444P, 8302Y, 8868R, 8301X.
