# Supplementary material for: Antipsychotic prescribing patterns in Australia: a retrospective analysis
Source: BMC Psychiatry. 2022 Feb 12;22:110. doi: 10.1186/s12888-022-03755-z (PMC8840322; doi:10.1186/s12888-022-03755-z)
Supplement: Supplementary file 2 — Additional file 2. [file 12888_2022_3755_MOESM2_ESM.docx]

**SUPPLEMENTAL TEXT**

**Ethical Considerations**

The Pharmaceutical Benefits Scheme (PBS), administered by the Australian Government, provides subsidised access to medicines. The PBS subsidises approximately 75% of prescribed medication use in Australia [1]. PBS/RPBS dispensing claims submitted for payment of a government subsidy are processed by the Department of Human Services (Services Australia) and are provided to the Department of Health and Department of Veterans affairs for monitoring, evaluation and health service planning [1]. Information collected at the time of claims processing forms the basis of the PBS data collection.

Unit-record formats for a subset of 10% of the Australian population are made available to contracted providers upon request to answer specific research questions. The PBS 10% sample is a de-identified, standardised, longitudinal, unit record-extract for a random 10% of the Australian population [1]. The Department of Health provides PBS data to approved providers. Under the Australian Government Department of Health privacy policy, personal information may be disclosed to individual researchers for ‘policy development, program evaluation, research, surveys (including one off and longitudinal) and reports of health activities and businesses’ [2].

The *Australian Government Department of Human Services External Request Evaluation Committee* functions essentially as an ethics committee and reviews purpose, anonymity, consent, secrecy, resource use, reputation , and other policies [3].  The aim of deidentification is to obscure identifiable data items with a persons’ records sufficiently that the risk of potential identification of the subject or a person’s record is minimised to acceptable level, and hence it is not required under Australian law to obtain individual informed consent [4].

Therefore, where it is impracticable to obtain an individual’s explicit consent to the use of their information and the purpose of the research cannot be served by using non-identifiable information, researchers must comply with the *Guidelines under Section 95 of the Privacy Act 1988* (s95 guidelines) or the *Guidelines approved under Section 95A of the Privacy Act 1988* (s95A guidelines) (as applicable) to ensure that their handling of personal information does not breach the *Privacy Act 1988*. Where researchers need approval to use an opt-out approach for research to which the s95 or 95A guidelines apply, only an HREC may grant this approval. Other review bodies may approve an opt-out approach for other research (Section 2.3, [4]).

Thus the process for access and publication of the data involves qualified vendors accessing the data under an agreement with Services Australia, and each individual research project must be approved by the Australian Government Department of Human Services External Request Evaluation Committee (EREC).  Formal HREC approval is only required where the PBS is being linked to other datasets.

In this study, the approved provider was Prospection Pty Ltd, and approval for this study was provided by Services Australia EREC, approval number RMS1280. Prospection Pty Ltd is listed as a commercial source of PBS information on the Australian Government Department of Health PBS website [5].

Studies of routinely collected data from the PBS 10% Sample provide insights into real-world medication use.

**References**

1. Mellish, L., et al., *The Australian Pharmaceutical Benefits Scheme data collection: a practical guide for researchers.* BMC Res Notes, 2015. **8**: p. 634.

2. Australian Government Department of Health, *Privacy Policy*. 2020.

3. Australian Government Services Australia. *Statistical information and data available from* [*https://www.servicesaustralia.gov.au/organisations/about-us/reports-and-statistics/statistical-information-and-data#a2*](https://www.servicesaustralia.gov.au/organisations/about-us/reports-and-statistics/statistical-information-and-data#a2). 2021.

4. National Health and Medical Research Council, The Australian Research Council, and Universities Australia, *National Statement on Ethical Conduct in Human Research (2007) Updated 2018.* 2018, Commonwealth of Australia: Canberra.

5. Australian Government Department of Health. *Sources of data for use in generating utilisation estimates availbale from* [*https://www.pbs.gov.au/info/industry/useful-resources/sources*](https://www.pbs.gov.au/info/industry/useful-resources/sources). 2020.
